# *Kingella negevensis* shares multiple putative virulence factors with *Kingella kingae*

**DOI:** 10.1371/journal.pone.0241511

**Published:** 2020-10-30

**Authors:** Eric A. Porsch, Pablo Yagupsky, Joseph W. St. Geme

**Affiliations:** 1 Department of Pediatrics, Children’s Hospital of Philadelphia, Philadelphia, Pennsylvania, United States of America; 2 Clinical Microbiology Laboratory, Soroka University Medical Center, Beer-Sheva, Israel; 3 University of Pennsylvania Perlman School of Medicine, Philadelphia, Pennsylvania, United States of America; Instituto Butantan, BRAZIL

## Abstract

*Kingella negevensis* is a newly described gram-negative bacterium in the Neisseriaceae family and is closely related to *Kingella kingae*, an important cause of pediatric osteoarticular infections and other invasive diseases. Like *K*. *kingae*, *K*. *negevensis* can be isolated from the oropharynx of young children, although at a much lower rate. Due to the potential for misidentification as *K*. *kingae*, the burden of disease due to *K*. *negevensis* is currently unknown. Similarly, there is little known about virulence factors present in *K*. *negevensis* and how they compare to virulence factors in *K*. *kingae*. Using a variety of approaches, we show that *K*. *negevensis* produces many of the same putative virulence factors that are present in *K*. *kingae*, including a polysaccharide capsule, a secreted exopolysaccharide, a Knh-like trimeric autotransporter, and type IV pili, suggesting that *K*. *negevensis* may have significant pathogenic potential.

## Introduction

The genus *Kingella* in the family Neisseriaceae contains the species *K*. *kingae*, *K*. *oralis*, *K*. *denitrificans*, *K*. *potus*, and the novel species *K*. *negevensis*. Of these species, *K*. *kingae* is the most common etiology of human disease, primarily causing osteoarticular infections and bacteremia in the pediatric population [1,2]. In the course of epidemiological studies examining carriage of *K*. *kingae* in the upper respiratory tract in healthy children in Israel, a small colony variant was identified with microbiological characteristics resembling *K*. *kingae* [3,4]. Further analysis of these isolates revealed significant differences relative to *K*. *kingae*, resulting in assignment to a novel species designated *K*. *negevensis* [4,5].

Due in part to the challenges of culture-based methods to detect *K*. *kingae* in clinical specimens, PCR-based diagnostic strategies targeting the *rtxA* and *rtxB* genes in the *rtx* (repeats-in-toxin) locus were developed [6–9]. Examination of the *K*. *negevensis* genome sequence revealed the presence of a highly homologous *rtx* locus, including highly homologous *rtxA* and *rtxB* genes that are amplified with the *K*. *kingae* primers. As a consequence, PCR assays that target *rtxA* and *rtxB* are unable to differentiate between *K*. *negevensis* and *K*. *kingae*, raising the possibility that some infections attributed to *K*. *kingae* might instead be due to *K*. *negevensis* [10,11]. To circumvent this problem, El Houmami *et al*. developed a novel PCR-based test targeting the *groEL* gene, which is distinct in *K*. *kingae* and *K*. *negevensis* and can distinguish between these species [11]. Using the *groEL* assay, analysis of specimens from 99 cases of culture-negative osteoarticular infections in children age 6 to 48 months revealed 41 *K*. *kingae* infections and one *K*. *negevensis* infection [11]. The only other infections due to K. negevensis reported to date include a case of vaginosis in a 22-year old woman [10] and a vision-threatening polymicrobial eye infection in a 38-year old [12]. The study by El Houmami and colleagues suggests that *K*. *negevensis* may be a less common pathogen than *K*. *kingae*, at least as a cause of osteoarticular infections.

In considering the pathogenic potential of *K*. *negevensis*, it is notable that analysis of the genome of *K*. *negevensis* strain *eburonensis* by Opota *et al*. revealed several genes with homology to putative virulence genes in *K*. *kingae*. In addition to the *rtx* locus, *K*. *negevensis* contains a gene encoding a homolog of the *K*. *kingae* Knh trimeric autotransporter and genes involved in type IV pilus biogenesis and polysaccharide capsule export [10]. Additionally, strain *eburonensis* encodes a predicted type Vb two-partner secretion system homologous to the *Bordetella pertussis* filamentous hemagglutinin (FHA) system, which is absent in *K*. *kingae* [10].

In this study, we sought to extend the earlier genomic analysis of *K*. *negevensis*, aiming to determine whether *K*. *negevensis* strains actually produce type IV pili, a Knh homolog, a polysaccharide capsule, and a secreted exopolysaccharide. Our results suggest that *K*. *negevensis* may have significant pathogenic potential.

## Results

### *K*. *negevensis* produces an exopolysaccharide

**Fig 1:**
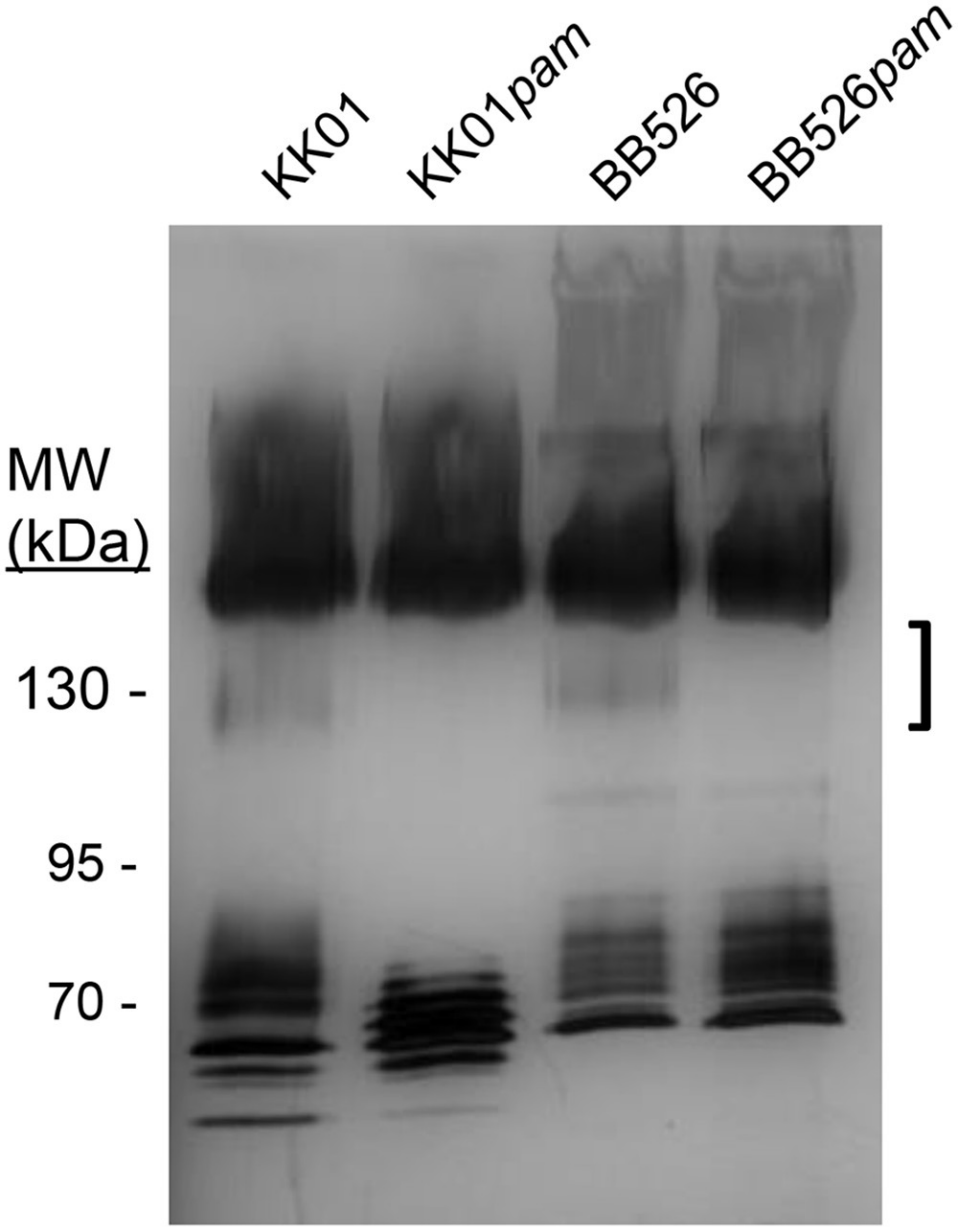
*K*. *negevensis* strain BB526 produces an exopolysaccharide. Heat extracts from control *K*. *kingae* strains KK01 and KK01*pam* along with *K*. *negevensis* strains BB526 and BB526*pam* were separated using SDS-PAGE and were then stained with silver. The exopolysaccharide material is evident in the bracketed region of the gel.

Genome analysis of *K*. *negevensis* revealed a locus with significant homology to the *pamABCDE* locus involved in production of a galactofuranose exopolysaccharide in *K*. *kingae* [13,14]. The homology between each predicted gene product and the *K*. *kingae* homolog is 99–100%. To determine whether *K*. *negevensis* produces an exopolysaccharide that is dependent on the *pamABCDE* locus, a *pamABC* deletion was created in strain BB526, generating strain BB526*pam*. Strains BB526 and BB526*pam* were subjected to a heat extraction to remove the exopolysaccharide, using *K*. *kingae* strains KK01 and KK01*pam* as controls. The resulting extracts were treated with proteinase K and were then separated on an SDS-PAGE gel and stained with silver. As shown in Fig 1, silver-stained material in the 100–130 kDa molecular weight range (see the bracketed area) was detected in strains KK01 and BB526 but was absent in stains KK01*pam* and BB526*pam*. These results indicate that *K*. *negevensis* strain BB526 produces an exopolysaccharide that is dependent on the *pamABCDE* locus.

### *K*. *negevensis* produces a polysaccharide capsule similar to the *K*. *kingae* type b capsule

**Fig 2:**
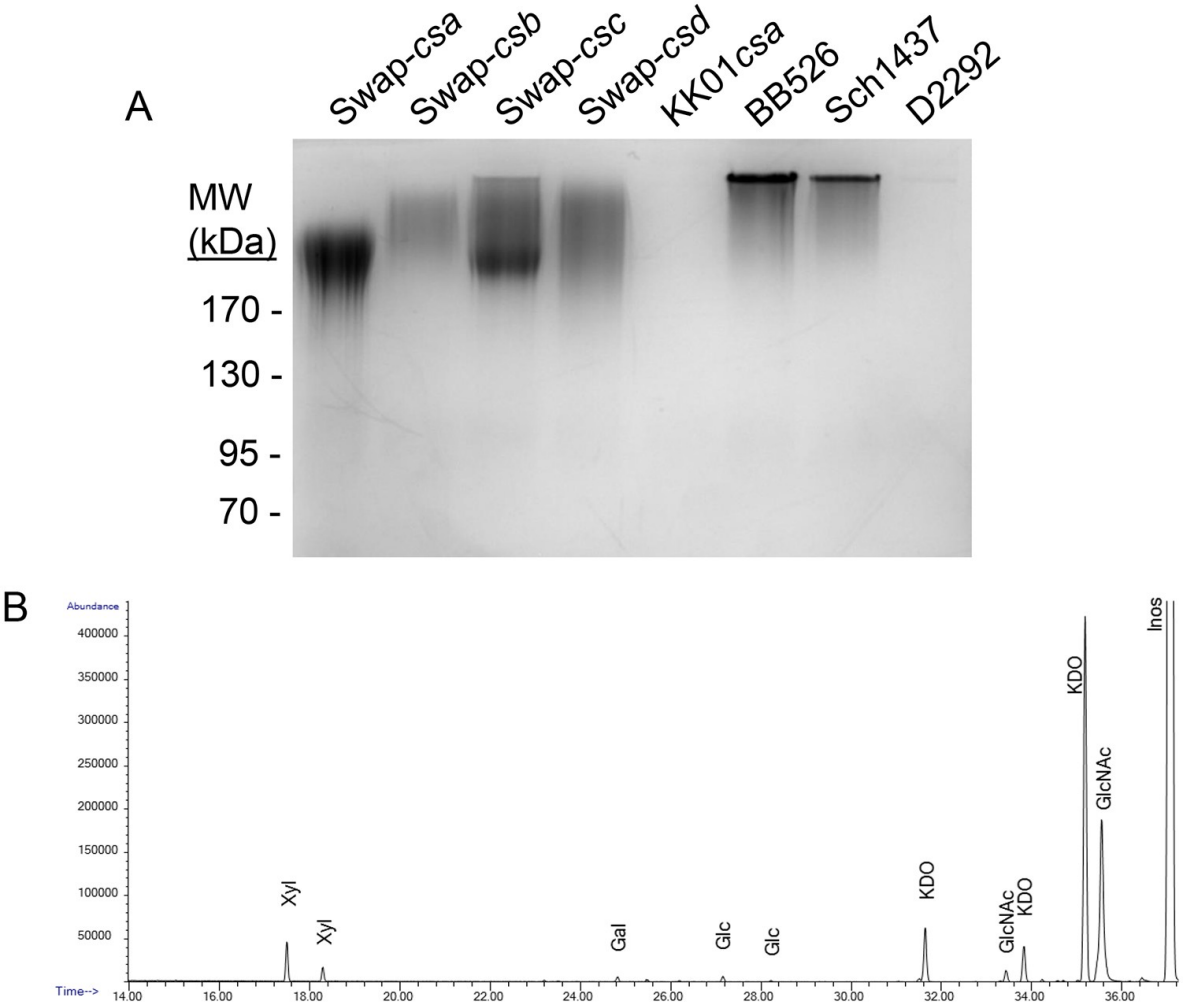
*K*. *negevensis* elaborates a polysaccharide capsule composed of N-acetylglucosamine (GlcNAc) and keto-deoxyoctulosonate (Kdo). **(A)** Surface acid extracts from control isogenic *K*. *kingae* strains Swap-*csa*, Swap*-csb*, Swap-*csc*, Swap-*csd*, and KK01*csaA* and *K*. *negevensis* strains BB526, Sch1437, and D2292 were separated using SDS-PAGE and were then stained with Alcian blue. The high molecular weight stained material is the capsular polysaccharide. **(B)** Combined gas chromatography/mass spectrometry (GC/MS) spectrum of the polysaccharide capsule isolated from *K*. *negevensis* strain BB526 revealed an abundance of GlcNAc and Kdo.

To determine whether *K*. *negevensis* produces a polysaccharide capsule, whole bacteria were treated with mild acid (tris-acetate, pH 5.0). The resulting extracts were treated with proteinase K, separated on an SDS-PAGE gel, and stained with the cationic dye Alcian blue. Isogenic *K*. *kingae* strains containing the four different capsule synthesis loci (capsule swap strains [15]) and a capsule synthesis mutant (KK01*csaA*) were used as controls. As shown in Fig 2A, high molecular mass Alcian blue-stained material indicative of capsular polysaccharide was observed in two of the three *K*. *negevensis* strains, similar to the four *K*. *kingae* isogenic capsule type control strains.

To begin to investigate whether there are multiple capsule types in the *K*. *negevensis* population, we began by searching the publicly available genomes for homologs of genes present in the four capsule synthesis loci in *K*. *kingae* [15]. All three available genomes were found to contain a putative capsule synthesis locus with homology to the *K*. *kingae csbA*, *csbB*, and *csbC* genes. The intra-species homology of each of these genes was high, with 99–100% identity of all three genes between the three strains with publicly available genome sequences, indicating that these strains likely produce the same capsule type. To extend this finding, we designed a PCR assay to detect the *csbA* gene and then examined our full panel of *K*. *negevensis* isolates (Table 1) with this assay. Using this assay, we found that all 19 of these isolates contain the *csbA* gene, indicating that all of these *K*. *negevensis* strains possess the same capsule synthesis locus. To explore the absence of capsule in the tris-acetate surface extract of *K*. *negevensis* strain D2292 (Fig 2A), we performed Sanger sequencing of the *ctrABCD* (capsule export), *lipAB* (capsule assembly), and *csbABC* (capsule synthesis) loci. This analysis revealed no deletion, insertion, or nonsense mutations in these loci, suggesting that a mutation(s) outside of the known capsule production genes is responsible for the capsule-deficient phenotype in this strain.

**Table 1:** Wild-type *K*. *negevensis* isolates used in this study.

Strain	PFGE complex
BB331	T
BB526	T
CC173	T
CC443	T
EPA014	T
PVC1712	T
D7641	b
AA503	b
CC505b	b
AA267	b
D7323	b
PED555	b
Sch538	ɳ
SW426	ɳ
Sch1437	ɳ
CC132	Unique 1
BB632	Unique 2
D2292	untypeable
EPA009	untypeable

*All isolates are from Houmami *et al*. [4].

To determine the glycosyl composition of the polysaccharide capsule from a representative *K*. *negevensis* strain, capsular material was extracted and purified from the surface of strain BB526*pam* by precipitation with the cationic detergent cetavlon (hexadecyltrimethylammonium bromide). We utilized the exopolysaccharide knockout mutant of strain BB526 to ensure that the sugars identified in the composition analysis were associated with the capsular polysaccharide and not the exopolysaccharide, consistent with the approach that we have used in the past to characterize *K*. *kingae* polysaccharide capsules [14,15]. As shown in Fig 2B, combined gas chromatography/mass spectrometry (GC/MS) of the per-O-trimethylsilyl (TMS) derivatives of the monosaccharide methyl glycosides produced from the sample by acidic methanolysis revealed that the capsule contains N-acetylglucosamine (GlcNAc) and keto-deoxyoctulosonate (Kdo), the same two sugars present in the *K*. *kingae* type b capsule.

### *K*. *negevensis* produces type IV pili

**Fig 3:**
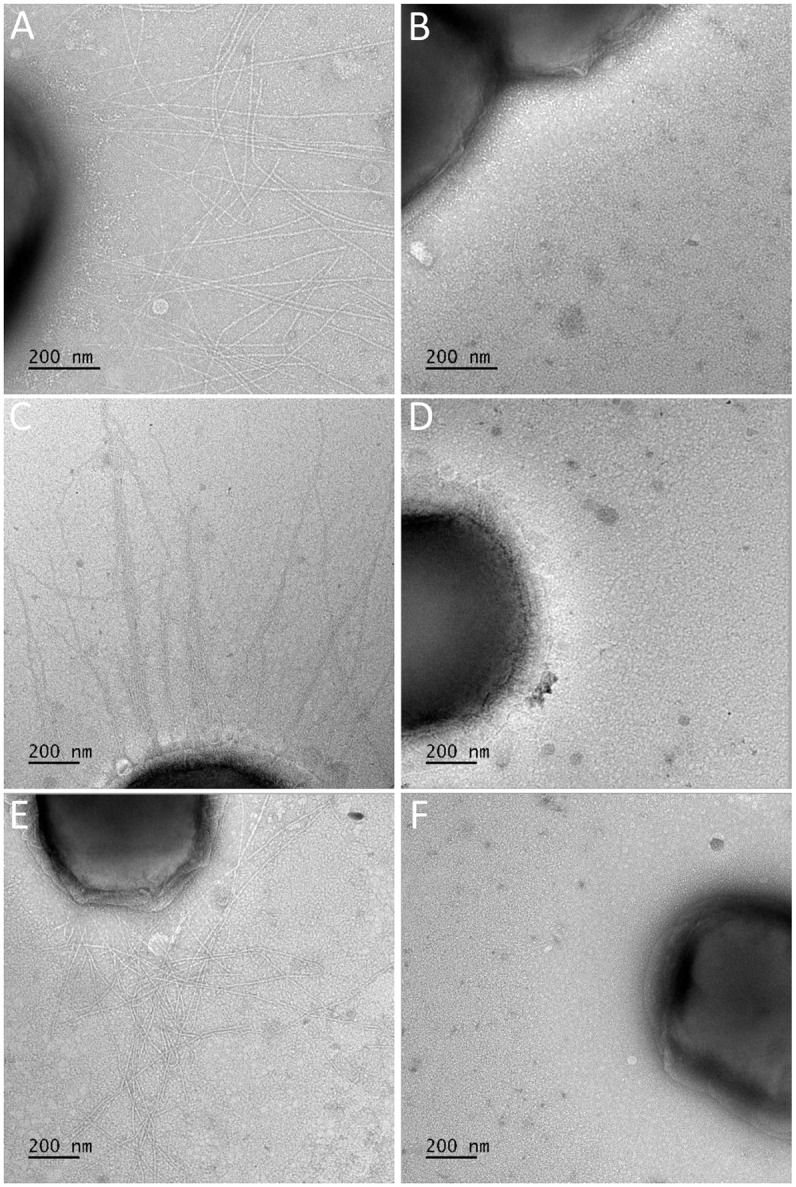
*K*. *negevensis* produce surface fibers. Strains BB526 **(A)**, BB526*pilA* (**B)**, Sch1437 **(C)**, Sch1437*pilA* **(D)**, D2292 **(E)**, and D2292*pilA* **(F)** were negatively stained and visualized using TEM. Abundant long surface fibers are present on the surface of the wild type strains BB526, Sch1437, and D2292 but are absent from the surface of each isogenic *pilA* mutant.

**Fig 4:**
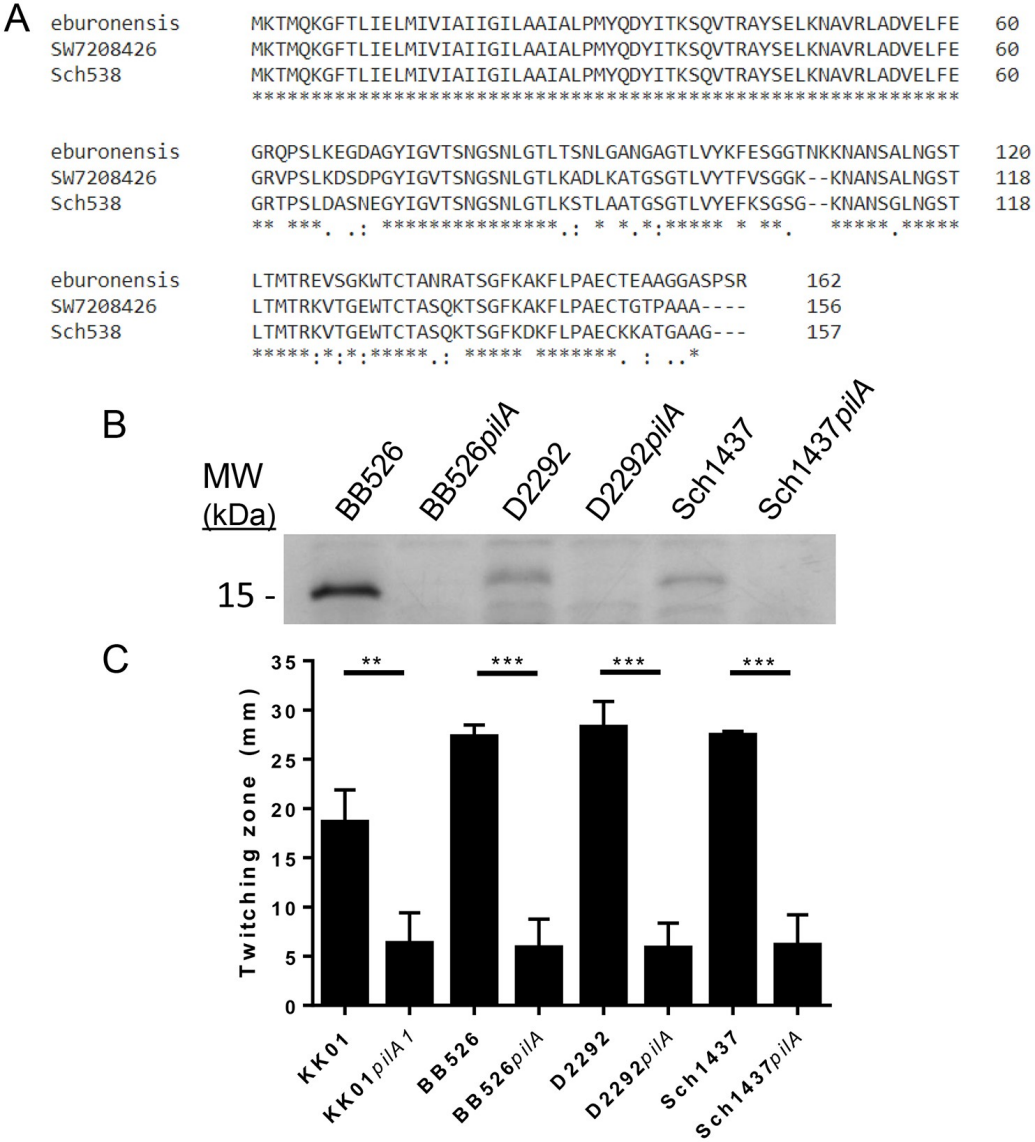
*K*. *negevensis* surface fibers are type IV pili capable of mediating twitching motility. **(A)** A multiple sequence alignment of the putative major pilin subunit from *K*. *negevensis* strains eburonensis, SW7208426, and Sch538 is shown. **(B)** Surface fibers were sheared from the bacterial surface, resolved by SDS-PAGE, and stained with Coomassie blue, revealing the major pilin subunit. The major pilin subunit band is evident in wild type strains BB526, D2292, and Sch1437 but is absent from the mutant strains BB526*pilA*, D2292*pilA*, and Sch1437*pilA*. **(C)** The twitching motility zones were for strains KK01, KK01*pilA1*, BB526, BB526*pilA*, D2292, D2292*pilA*, Sch1437, and Sch1437*pilA* were determined. The twitching zone diameter for each wild type and isogenic *pilA* mutant strain were shown to be significantly different using student’s T-test. (**p<0.005, ***p<0.0005).

Genome analysis by Opota *et al*. revealed the presence of several genes associated with type IV pilus biogenesis in *K*. *negevensis* strain *eburonensis* [10]. To begin to investigate whether *K*. *negevensis* produces surface pili, strains BB526, Sch1437, and D2292 were examined by negative staining transmission electron microscopy (TEM). We selected these three strains because we have demonstrated that they are naturally competent and thus are amenable to genetic manipulation. As shown in Fig 3A, 3C and 3E, all three strains had surface fibers that were visible using negative staining TEM. To confirm that these are type IV pilus fibers, we searched the available genomes for a putative type IV major pilin, the primary structural unit of type IV pili. Consistent with the fact that species that produce type IV pili typically possess multiple pilin-like genes representing the major pilin and multiple minor pilins, we were able to identify multiple pilin-like genes. In *K*. *kingae*, there is significant amino acid sequence diversity in the major pilin subunit and much greater conservation in the minor pilins among diverse strains [16]. To identify the major pilin subunit bioinformatically, we hypothesized that the pilin-like gene with the greatest sequence diversity among *K*. *negevensis* isolates was the most likely candidate. We identified one pilin-like gene that ranged in sequence homology from 79.6% identity/86.4% similarity to 87.3% identity/88.5% similarity in pairwise comparisons, contrasting with all of the other pilin-like genes, which had at least 99% identity. Fig 4A shows a multiple alignment of the predicted amino acid sequences of this gene product from strains *eburonensis*, SW7208426, and Sch538. In keeping with the nomenclature in *K*. *kingae*, we designated this gene *pilA*. We next generated a knockout of *pilA* in strains BB526, Sch1437, and D2292 and examined the resulting mutants by negative staining TEM. As shown in Fig 3B, 3D and 3F, strains BB526*pilA*, Sch1437*pilA*, and D2292*pilA* all lacked visible surface fibers, suggesting elimination of a critical type IV pilus biogenesis factor. To extend these results, the wild type and mutant strains were subjected to vortexing and ammonium sulfate precipitation to recover surface fibers. The resulting samples were resolved on an SDS-PAGE gel and stained with Coomassie blue. As shown in Fig 4B, a major band with a molecular mass of approximately 15 kDa was present in the three wild type strains and absent from the isogenic *pilA* mutants (Fig 4B). These results suggest that the mutant strains lack the major pilin subunit.

Twitching motility is a form of surface motility that results from the extension and retraction of type IV pili. To determine if *K*. *negevensis* type IV pili are capable of mediating this process, bacteria were stab inoculated to the Petri plate-agar interface and incubated for 2 days as described previously [17]. Twitching motility is evident when there is spread of growth from the central inoculation site. As shown in Fig 4C, strains BB536, D2292, and Sch1437 produced similar twitching zone sizes, while the non-piliated mutant strains BB526*pilA*, D2292*pilA*, and Sch1437*pilA* produced statistically significant smaller zones (*p*<0.0005 for all comparisons between wild type and isogenic *pilA* mutant), on par the with KK01*pilA1* non-piliated control strain. Taken together, these data indicate that *K*. *negevensis* produces type IV pili capable of mediating twitching motility.

### *K*. *negevensis* produces a trimeric autotransporter homologous to *K*. *kingae* Knh

**Fig 5:**
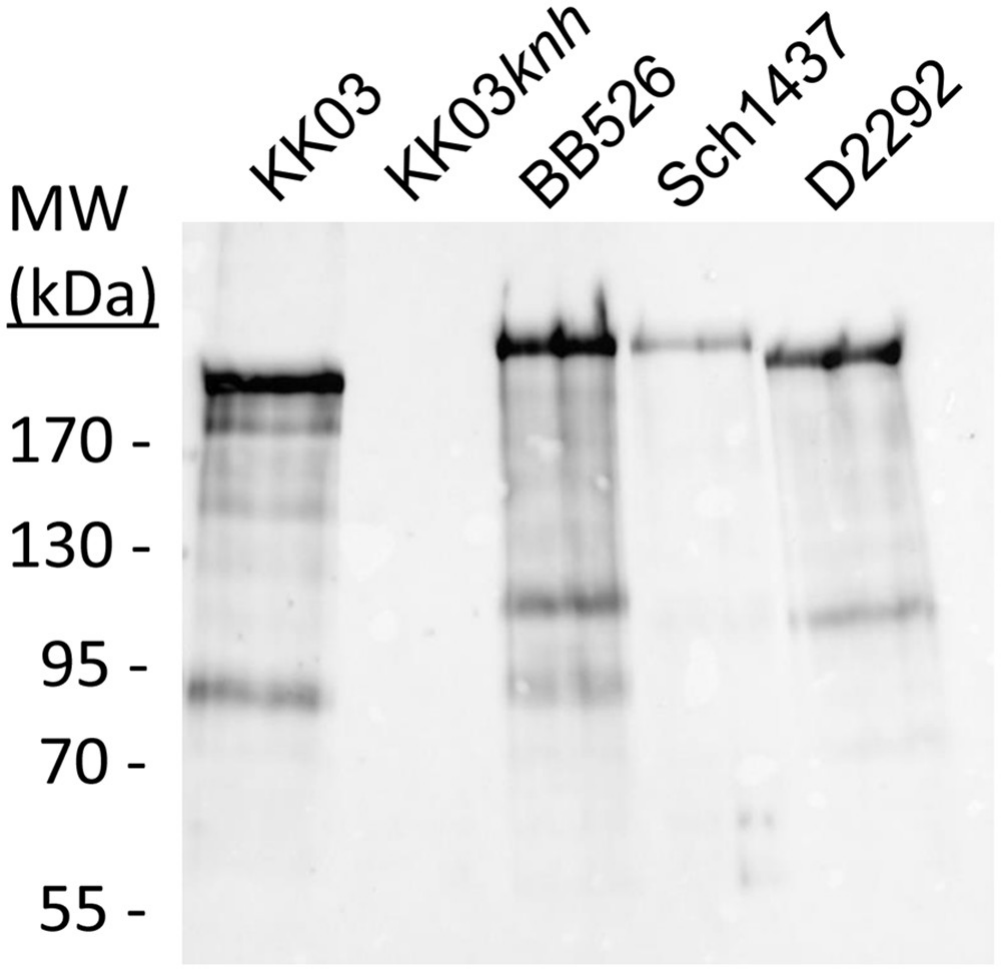
*K*. *negevensis* produces a Knh homolog. Outer membrane fractions were isolated from strains KK03, KK03*knh*, BB526, Sch1437, and D2292, treated with formic acid, and separated using SDS-PAGE. Following transfer to nitrocellulose, samples were probed with GP97, an antiserum targeting the YadA-like head domains of *K*. *kingae* Knh. All three *K*. *negevensis* strains have a high molecular weight reactive band, indicating production of the Knh homolog, similar to the positive control *K*. *kingae* KK03.

Genome analysis revealed the presence of a gene encoding a predicted trimeric autotransporter with homology to the *K*. *kingae* Knh protein, with 36.1% identity/44.7% similarity between *K*. *kingae* strain KK03 and *K*. *negevensis* strain Sch538. The regions in these proteins with the highest levels of homology are the N-terminal YadA-like head domain region and the C-terminal beta-barrel region. To determine whether *K*. *negevensis* produces this predicted Knh-like protein, outer membranes fractions from strains BB526, Sch1437, and D2292 were isolated, denatured with formic acid, and separated using SDS-PAGE. Following transfer to nitrocellulose, samples were probed with GP97, an antiserum that was generated against the *K*. *kingae* Knh YadA-like head domain [17]. As shown in Fig 5, strains BB526, Sch1437, and D2292 were reactive with GP97, yielding bands in the high molecular mass range consistent with the predicted molecular masses. *K*. *kingae* strain KK03 and an isogenic *knh* mutant were used as controls. This result indicates that *K*. *negevensis* produces a protein homologous to the *K*. *kingae* Knh trimeric autotransporter protein.

## Discussion

The recent recognition of *K*. *negevensis* as a novel distinct species in the *Kingella* genus has raised several important questions about putative virulence factors expressed by this organism. Our data show that *K*. *negevensis* elaborates a secreted exopolysaccharide and a polysaccharide capsule similar to the GlcNAc-Kdo capsule of type b-encapsulated *K*. *kingae*. In addition, *K*. *negevensis* produces type IV pili and a high-molecular mass adhesin similar to the *K*. *kingae* Knh trimeric autotransporter protein.

It is intriguing that all of the *K*. *negevensis* isolates analyzed here contain the same capsule synthesis locus, indicating that they elaborate the same capsule polysaccharide structure. In contrast, the *K*. *kingae* population contains four distinct capsule types [15]. It should be noted that >600 *K*. *kingae* isolates from invasive disease cases and healthy carriers worldwide have been analyzed to date [15,18], while only 19 isolates of *K*. *negevensis* (18 from Israel and one from Switzerland) were examined for capsule type in this study. Sequence analysis from publicly available genomes of two European isolates (SW7208426 and *eburonensis*) reveals the same capsule synthesis locus as is present in the 19 isolates analyzed here. While there are different pulse field gel electrophoresis (PFGE) groups represented in these isolates [4], the small number and limited geographic range of the isolates make it difficult to know whether there truly is only one capsule type in this species.

As type IV pili and Knh are putative adhesive factors, it likely that these surface structures promote adherence to respiratory epithelial cells, as has been shown for *K*. *kingae* [17,19]. Interestingly, there are significant differences in the genetic arrangement and content of type IV pilus genes in the two species. While the *K*. *kingae* pilin locus contains the major pilin subunit gene *pilA1* and two minor pilin-like genes, *pilA2* and *fimB* [19,20], the *K*. *negevensis* pilin locus contains only one pilin-like gene, designated *pilA*. *K*. *kingae* type IV pili contain two pilus-associated proteins called PilC1 and PilC2, at least one of which must be produced to allow type IV pilus biogenesis and type IV pilus-mediated phenotypes [19,21]. Analysis of the *K*. *negevensis* genome reveals a single *pilC* gene, which encodes a predicted protein with limited homology to *K*. *kingae* PilC2 (34.7% identity/49.3% similarity).

Yagupsky *et al*. examined healthy children in Israel for carriage of *K*. *negevensis* and found carriage rates approximately 1/10^th^ the rate of *K*. *kingae* carriage [22]. Only culture-based methods were deployed in this study, raising the possibility that *K*. *negevensis* colonization was missed in some children, either because of low density colonization or because of inefficient recovery by culture [22]. Future studies of pharyngeal carriage in young children across diverse geographic locations utilizing the PCR-based detection method capable of distinguishing *K*. *negevensis* from *K*. *kingae* developed by El Houmami *et al*. are necessary to define the true carriage rate of *K*. *negevensis* [11]. Combining PCR-based and culture-based sampling would provide even greater information, allowing recovery of viable organisms for additional analyses.

It is interesting to consider the possibility that *K*. *negevensis* colonizes the respiratory tract less efficiently and causes disease less frequently than *K*. *kingae*. While the two organisms produce similar putative virulence factors, including a secreted exopolysaccharide, a polysaccharide capsule, type IV pili, and Knh, they differ in morphology. *K*. *negevensis* appears as long chains, while *K*. *kingae* typically appears as pairs or short chains [4]. The longer chain length may make it more difficult for the bacteria to breach the respiratory epithelial barrier and enter the bloodstream, reducing the likelihood that distal sites of infection will be seeded. The longer chain length may also facilitate mucociliary clearance. In addition, both organisms encode a secreted RTX toxin, but some *K*. *kingae* strains have two copies of the toxin gene *rtxA* whereas the available *K*. *negevensis* genome sequences reveal only one *rtxA* gene copy [10]. This difference may lead to reduced production of RtxA relative to *K*. *kingae* and thus reduced damage to the respiratory epithelium. Future studies examining *K*. *negevensis* pathogenicity may reveal additional factors that temper this organism’s virulence compared to *K*. *kingae*.

It is intriguing that the study by El Houmami *et al*. suggests that *K*. *negevensis* may be less virulent than *K*. *kingae*, in particular given our findings that *K*. *negevensis* and *K*. *kingae* produce a number of the same putative virulence factors, raising the possibility that *K*. *negevensis* has significant pathogenic potential. One possibility is that *K*. *negevensis* infections are underrecognized due to the difficulty in recovering viable bacteria from clinical samples, a known issue with *Kingella* spp. While osteoarticular infections are the most common presentation of *K*. *kingae* infections, *K*. *negevensis* may cause other clinical conditions that are classified as culture-negative due to failure to recover or detect the organism. Future studies using molecular diagnostic approaches capable of detecting *K*. *negevensis* are necessary to address this possibility.

## Materials and methods

### Bacterial strains and plasmids

The bacterial strains used in this study are listed in Tables 1 and 2. The *K*. *negevensis* strains used in this study were previously collected from the upper respiratory tract of healthy children [3,4]. Eighteen of the isolates are from Israel and one is from Switzerland (SW426). The 19 *K*. *negevensis* strains are epidemiologically unrelated and represent a wide array of distinct PFGE clones. *K*. *kingae* strain KK03 is a stable spreading/corroding colony type, and KK01 is a stable nonspreading/noncorroding colony type of clinical isolate 269–492, which was recovered from the knee joint of a child with septic arthritis [23]. *K*. *kingae* strains were cultured at 37°C with 5% CO_2_ on chocolate agar, and *K*. *negevensis* strains were cultured at 37°C with 5% CO_2_ on brain heart infusion (BHI) agar supplemented with 10% sheep blood. Strains KK01*pilA1* [19], KK03*knh* [17], KK01*csaA* [24] were generated as previously described. Strain BB526*pam* was generated via natural transformation. Briefly, 250 ng of plasmid pUC19*pamABC*::*ermC* [14] was linearized with NdeI and mixed with 250 μl of a ~0.5 OD_600_ suspension of strain BB526 in BHI broth. After 30 minutes at ambient temperature, 250 μl of 20% lysed horse blood in BHI broth was added, and the reaction was incubated at 37°C with 5% CO_2_ for 2 hours prior to plating on chocolate agar containing 1 μg/mL erythromycin. Plasmid pUC19*pilA*::*aphA3* was generated by amplifying fragments corresponding to the surrounding 5′ and 3′ regions of the suspected *pilA* major pilin subunit gene were amplified using the primers pilA5′for (5’-AGCTGAATTCCGTAAAGTTCAATATCTTGCCCG-3’), pilA5′rev (5’-AGCTGGTACCTTGCATAGTTTTCATGTGTTTTATCTC-3’), pilA3′for (5’-AGCTGGATCCTGCTGGCTAAGGTTAAATCTAAAC-3’), and pilA3′rev (5’-ACGTAAGCTTGAACAAGGCGTGTCTTTGTG-3’), respectively. The *aphA3* kanamycin resistance cassette was amplified from plasmid pFalcon2 with flanking BamHI sites and was ligated between the 5’ and 3’ regions, generating pUC19*pilA*::*aphA3*. Strains BB526*pilA*, D2292*pilA*, and Sch1437*pilA* were generated as described above using natural transformation. All mutant strains were confirmed by PCR and Sanger sequencing. All *K*. *kingae* and *K*. *negevensis* strains were stored at -80°C in BHI broth with 20% glycerol, and all *E*. *coli* strains were stored at -80°C in LB broth with 15% glycerol.

**Table 2:** *K*. *kingae* and *K*. *negevensis* mutant strains used in this study.

Strain	Description	Source
KK03	Spreading/corroding *Kingella kingae* derivative of strain 269–492	[23]
KK01	Nonspreading/noncorroding *K*. *kingae* derivative of strain 269–492	[23]
KK01*pilA1*	KK01 with an *aphA3* disruption in *pilA1*	[19]
KK01*pam*	KK01 with an *ermC*-marked deletion of *pamABCDE*	[14]
KK01*csaA*	KK01 with *aphA3-*marked deletion of *csaA*	[24]
KK03*knh*	KK03 with a *tetM-*marked deletion of *knh*	[17]
KK01swap*csa*	KK01 expressing the type a capsule synthesis gene locus *csa*	[15]
KK01swap*csb*	KK01 expressing the type b capsule synthesis gene locus *csb*	[15]
KK01swap*csc*	KK01 expressing the type c capsule synthesis gene locus *csc*	[15]
KK01swap*csd*	KK01 expressing the type d capsule synthesis gene locus *csd*	[15]
BB526*pam*	BB526 with *ermC-*marked deletion of the *pamABC* genes	This study
BB526*pilA*	BB526 with *aphA3*-marked deletion of *pilA*	This study
D2292*pilA*	D2292 with *aphA3*-marked deletion of *pilA*	This study
Sch1437*pilA*	Sch1437 with *aphA3*-marked deletion of *pilA*	This study.

### Exopolysaccharide analysis

Bacteria were swabbed from BHI/10% sheep blood agar plates and suspended in 2 ml of 1 x PBS to an OD_600_ of 0.8. The bacteria were pelleted by centrifugation, resuspended in 100 μl 1 x PBS, and heated at 55°C for 30 minutes. The bacteria were again pelleted by centrifugation, and the supernatant was treated for 1 hr with 20 μg proteinase K at 55°C. The samples were separated on 7.5% SDS-PAGE gels and were stained with silver as previously described [14].

### Capsule analysis

For capsule typing analysis by PCR, the sequence of the putative capsule synthesis gene *csbA* from strain Sch538 was used as the template to design primers *csbA* F (5’-TCTCCGCGATTGTGGATTAC-3’) and *csbA* R (5’-ATAGGGCAAGCGTTCATAGG-3’). The resulting amplification produced a ~500 bp amplicon. For capsule staining, tris-acetate pH 5.0 capsule extractions were performed as previously described [17]. The extracts were separated on 7.5% SDS-PAGE gels and stained with 0.125% Alcian blue in 40% methanol/5% acetic acid [17].

For large scale capsule extractions, the method described by Jennings and Yang based on cetavlon precipitation was utilized with some modifications [25]. As *K*. *negevensis* does not grow well in liquid culture, strain BB526*pam* was inoculated onto 40 BHI plates supplemented with 10% sheep blood and was incubated for 17–18 hours at 37°C in a humidified 5% CO_2_ atmosphere. The bacterial growth was then swabbed into 250 mL of BHI broth, paraformaldehyde was added to a final concentration of 1%, and the suspension was shaken at 200 rpm for 30 min. After centrifugation at 8,000 x g for 30 min, the supernatant was subjected to 1% cetavlon precipitation at 4°C overnight. The precipitate was collected by centrifugation and was dissolved in 0.9 M CaCl_2_ and then subjected to 25% ethanol precipitation. The supernatant was retained and subjected to 80% ethanol precipitation. The precipitate was dissolved in 25 ml 0.2 M sodium phosphate pH 7.0 and extracted with an equal volume of phenol. The phenol phase was extracted a second time with 25 ml 0.2 M sodium phosphate buffer, and the aqueous phases were combined. After extensive dialysis in DI water, the retentate was flash frozen and lyophilized. The lyophilized material was then dissolved in 1 x PBS and treated with DNAse I, RNAse A, and proteinase K prior to separation on a 200 pg 16/600 gel filtration column. Fractions were analyzed by SDS-PAGE and Alcian blue staining to identify fractions containing capsule. Those fractions were pooled and lyophilized prior to further analysis.

Glycosyl composition analysis was performed at the University of Georgia Complex Carbohydrate Research Center (CCRC) by combined gas chromatography/mass spectrometry (GC/MS) of the per-O-trimethylsilyl (TMS) derivatives of the monosaccharide methyl glycosides produced from the sample by acidic methanolysis [26]. Briefly, the sample (100–200 μg) was heated with methanolic HCl in a sealed screw-top glass test tube for 18 h at 80°C. After cooling and removal of the solvent under a stream of nitrogen, the sample was treated with a mixture of methanol, pyridine, and acetic anhydride for 20 min for re-N-acetylation of amino sugars. After evaporation, the sample was derivatized with Sylon HTP^®^ (Sigma) at 80°C for 30 min. GC/MS analysis of the TMS methyl glycosides was performed on an Agilent 7890A GC interfaced to a 5975C MSD, using a Supelco Equity-1 fused silica capillary column (30 m x 0.25 mm ID).

### Pilus preparations

Pilus fractions were prepared by vortexing and ammonium sulfate precipitation as described previously [21]. The pilus fractions were separated on 15% SDS-PAGE gels and were stained with Coomassie blue.

### Negative staining transmission electron microscopy

Bacteria were swabbed from a plate and suspended to an OD_600_ of ~0.3 in 0.2 M ammonium acetate. Bacteria were allowed to bind to a 300 mesh carbon-coated copper grid for 1 minute. The grid was then wicked to dry with filter paper and placed on a drop of UranyLess negative staining solution (Electron Microscopy Sciences, Hatfield, PA) for 1 minute, wicked with filter paper, and allowed to dry. The bacteria were imaged using a FEI Tecnai 12 electron microscope at an accelerating voltage of 100 kV.

### Twitching motility assays

Bacteria were swabbed from BHI/10% sheep blood agar and suspended to an OD_600_ of ~0.8 in BHI broth. A 1 μl volume of the bacterial suspension was stab inoculated to the Petri dish-agar interface in a 100 mm tissue culture-treated Petri dish containing 15 ml BHI/10% sheep blood agar. The inoculated dishes were cultured for 48 hours at 37°C in a 5% CO_2_-enriched, humidified atmosphere. The agar was then carefully peeled away, and the zone of bacterial spread from the central inoculation was stained with 0.1% crystal violet prior to measurement.

### Western blotting

To detect Knh expression, outer membrane fractions were isolated based on sarkosyl insolubility and treated with formic acid as previously described [17]. The outer membranes were then separated on 7.5% SDS-PAGE gels, transferred to nitrocellulose, and probed with GP97 to detect Knh monomers [17].

## Supporting information

S1 File Raw gel and blot images. (PDF)
