# Supplementary figures and images for: Kingella negevensis shares multiple putative virulence factors with Kingella kingae
Source: PLoS One. 2020 Oct 30;15(10):e0241511. doi: 10.1371/journal.pone.0241511 (PMC7598479; doi:10.1371/journal.pone.0241511)

Fig 1 – Raw gel image

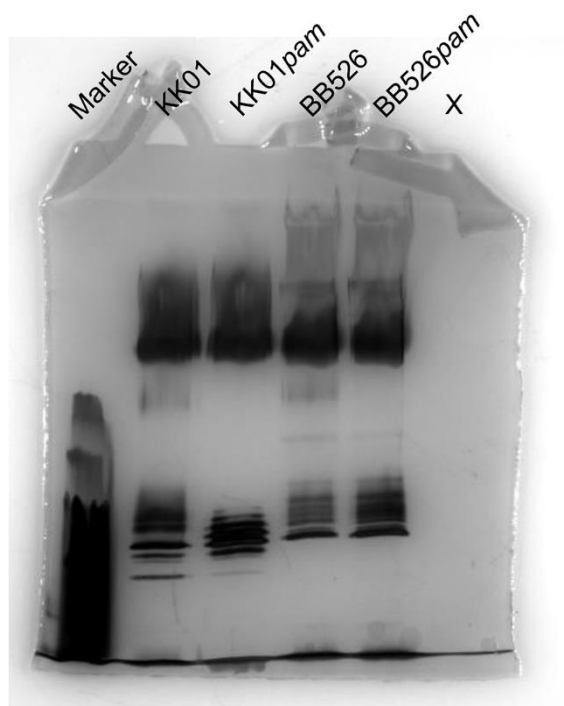

Fig 2A – Raw gel image

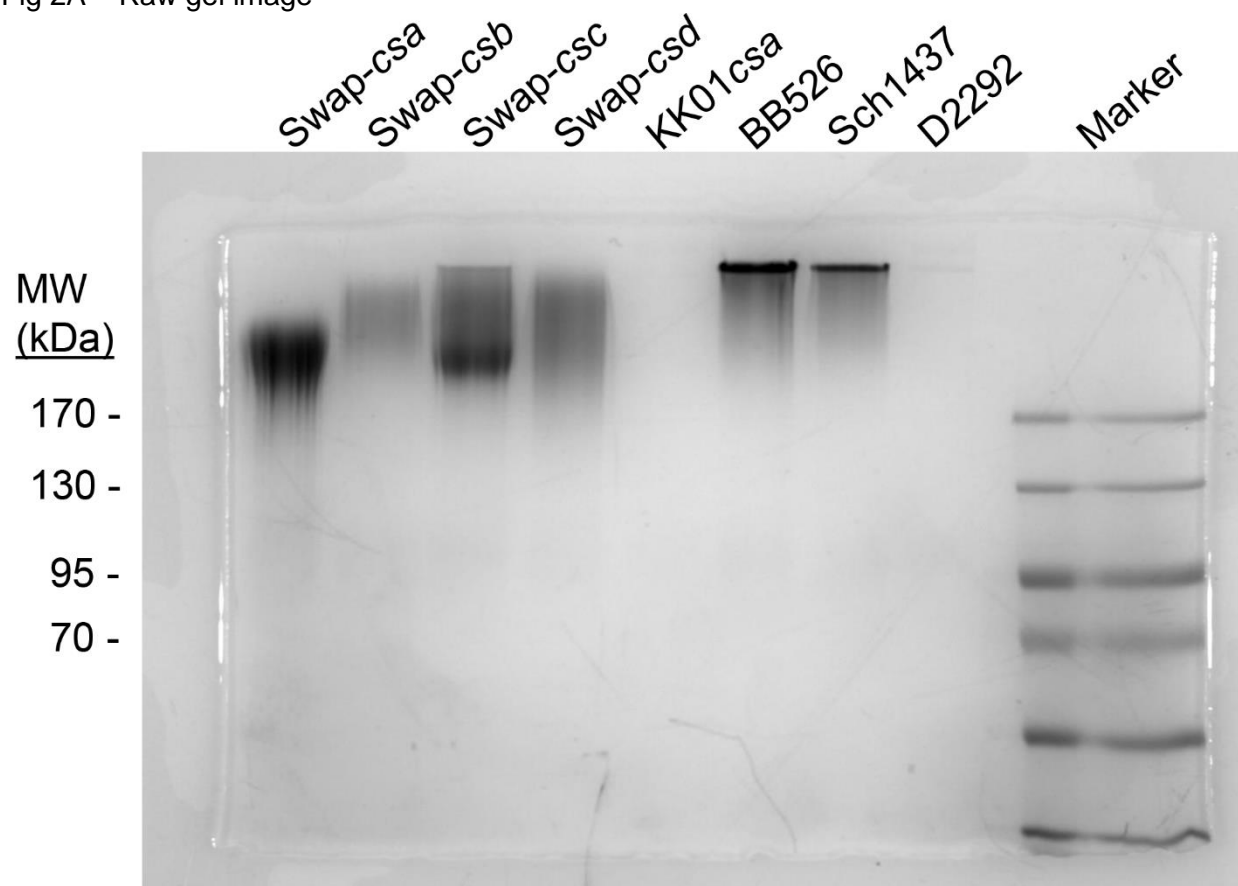

Fig 4B – Raw gel image

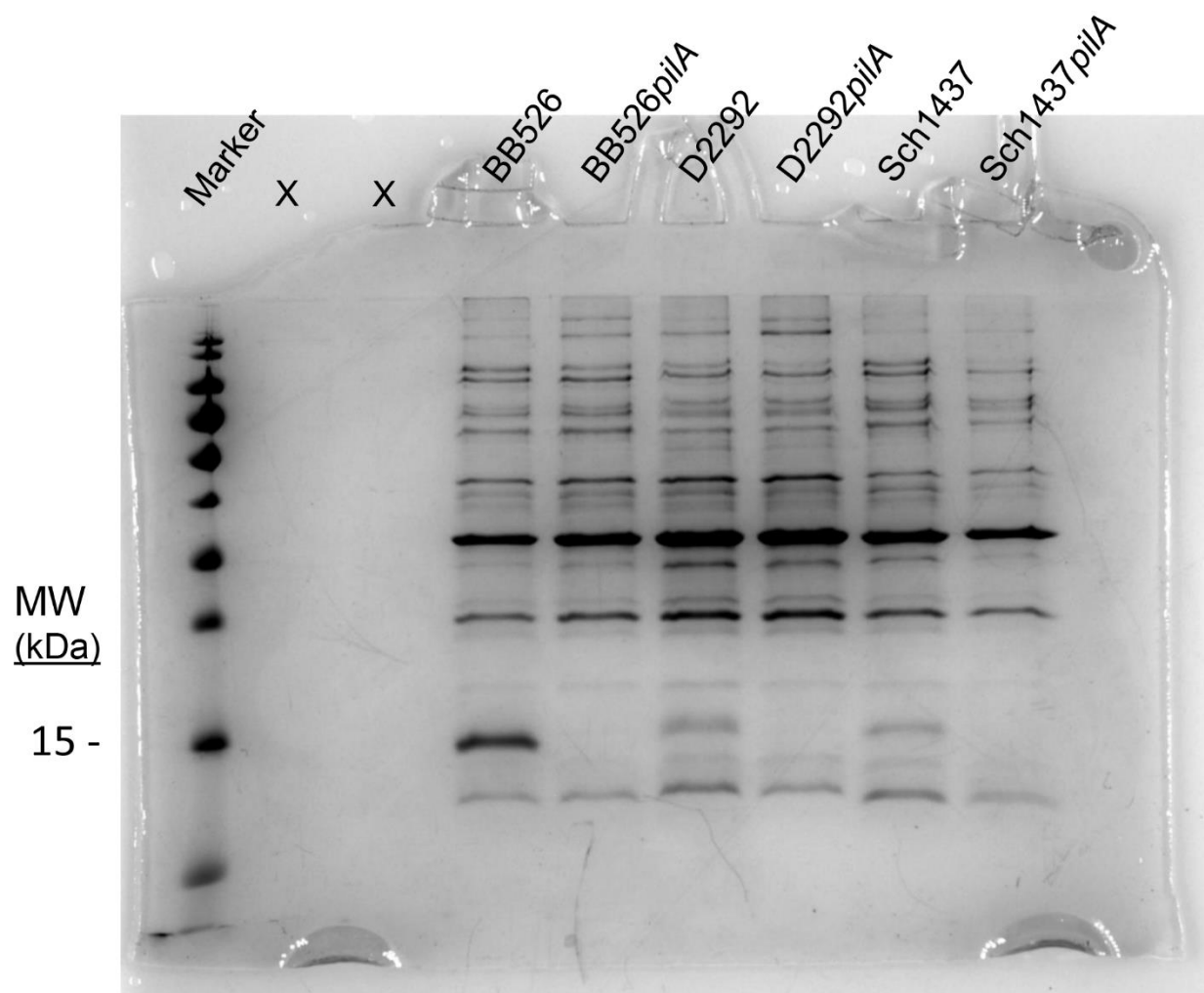

Fig 5 – Raw blot image

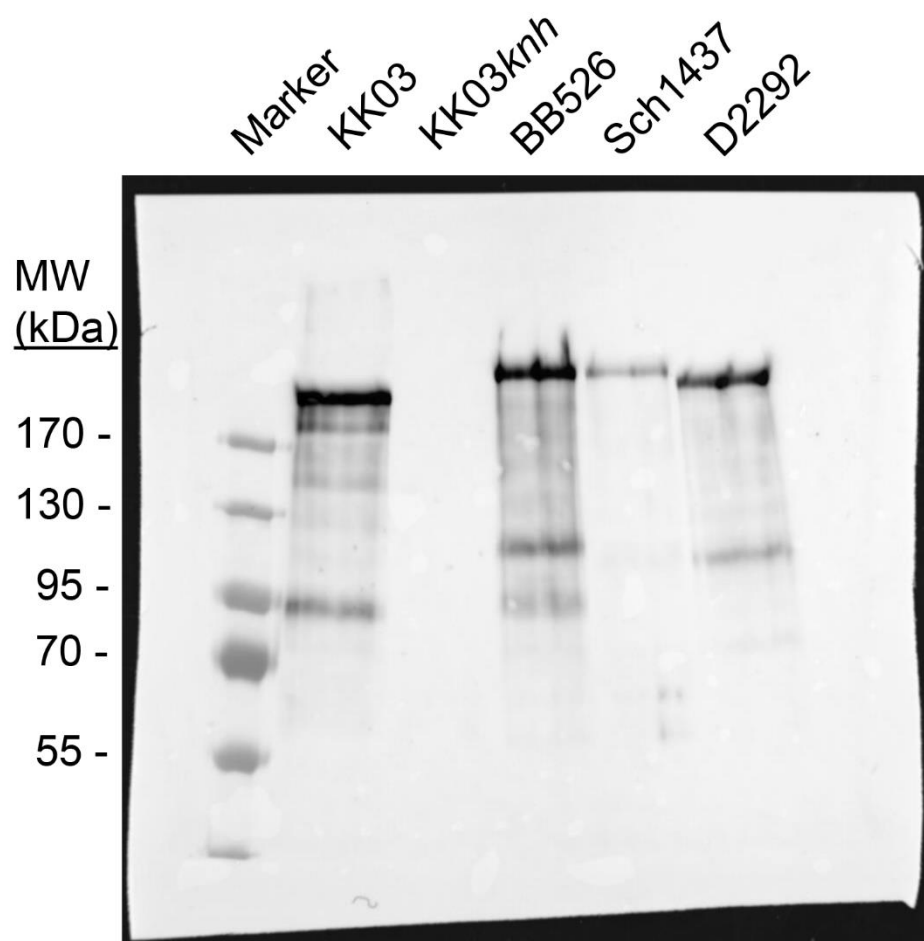

Supplement: S1 File — (PDF) [file pone.0241511.s001.pdf]
